# Potential non-invasive biomarkers in tumor immune checkpoint inhibitor therapy: response and prognosis prediction

**DOI:** 10.1186/s40364-023-00498-1

**Published:** 2023-06-02

**Authors:** Ruixia Song, Fengsen Liu, Yu Ping, Yi Zhang, Liping Wang

**Affiliations:** 1 Biotherapy Center and Cancer Center, The First Affiliated Hospital of Zhengzhou University, Zhengzhou, Henan China; 2 Department of Oncology, The First Affiliated Hospital of Zhengzhou University, Zhengzhou, Henan China; 3 Henan Key Laboratory for Tumor Immunology and Biotherapy, Zhengzhou University, Zhengzhou, Henan China; 4 School of Life Sciences, Zhengzhou University, Zhengzhou, Henan China; 5 State Key Laboratory of Esophageal Cancer Prevention & Treatment, Zhengzhou, Henan China

**Keywords:** Cancer, Immune checkpoint inhibitors, Biomarkers, Treatment outcome, Prognosis

## Abstract

Immune checkpoint inhibitors (ICIs) have dramatically enhanced the treatment outcomes for diverse malignancies. Yet, only 15–60% of patients respond significantly. Therefore, accurate responder identification and timely ICI administration are critical issues in tumor ICI therapy. Recent rapid developments at the intersection of oncology, immunology, biology, and computer science have provided an abundance of predictive biomarkers for ICI efficacy. These biomarkers can be invasive or non-invasive, depending on the specific sample collection method. Compared with invasive markers, a host of non-invasive markers have been confirmed to have superior availability and accuracy in ICI efficacy prediction. Considering the outstanding advantages of dynamic monitoring of the immunotherapy response and the potential for widespread clinical application, we review the recent research in this field with the aim of contributing to the identification of patients who may derive the greatest benefit from ICI therapy.

## Introduction

Over the past twelve years, immune checkpoint inhibitor (ICI) therapy has revolutionized the treatment landscape in oncology, with ipilimumab (binding to cytotoxic-T-lymphocyte-associated antigen 4 reported to remarkably improve the survival outcomes of patients with tumors [1]. As a relatively emerging therapy, in contrast with chemotherapy, ICI therapy has better therapeutic targeting, longer-lasting efficacy, and fewer systemic adverse effects [2]; moreover, compared with targeted therapy, it has wider applicability and is not limited to patients with tumors with specific mutations [3]. Owing to the better long-term efficacy in advanced or refractory tumors [4], basic and clinical research on ICI tumor therapy has been attracting increasing attention. By blocking the inhibitory immune signaling pathways in the tumor microenvironment (TME) [5], ICIs can avoid the apoptosis of T cells and rescue the cytotoxicity of tumor-specific T cells in the tumor microenvironment (TME); hence, they overcome the immunosuppressive environment, and effective anti-tumor response can be re-established [6–9].

Despite the remarkable breakthrough of ICI therapy in malignancy, only 15–60% of patients respond [10]. The major determinants contributing to ICI response and resistance are tumor-intrinsic factors [11, 12]. In addition, the landscape of tumor and TME can be shaped in the course of ICI treatment [13]. Indiscriminate administration of ICIs without systematic evaluation and selection may cause inappropriate or delayed treatment and wastage of considerable social and medical resources. Therefore, the exploration of biomarkers for ICI response and prognosis prediction has become a critical issue to be resolved. The US Food and Drug Administration-approved markers to predict ICI efficacy in the clinic include PD-L1 expression, mismatch repair-deficient/microsatellite instability-high, and tumor mutation burden (TMB). However, many other biomarkers with high predictability have also been validated [14–18]. Nevertheless, samples for these biomarkers have typically been obtained via invasive surgery or biopsy. Owing to the temporal and spatial complexity of the tumors and TMEs [19, 20], the biopsy sample from a single site does not seem to be representative of the overall landscape. Compared with invasive biomarkers, the novel non-invasive biomarkers have substantial advantages. First, as samples are usually acquired with no or minimal invasiveness, the possibility of tumor metastasis caused by the sample collection is eliminated and latent risks are avoided. Second, multiple non-invasive biomarkers have been demonstrated to systemically and accurately reflect disease status and overcome the spatial heterogeneity of tumor. Third, non-invasive samples are easily collected multiple times during ICI treatment, helping overcome tumor temporal heterogeneity and enabling dynamic and continuous monitoring of disease evolution. Fourth, non-invasive detection requires relatively fewer resources and less time, making it more accessible to patients and improving medical efficiency and effectiveness. Owing to the remarkable advantages described above, non-invasive markers show great potentiality to be widely applied to predict ICI efficacy in clinical practice.

**Fig. 1:**
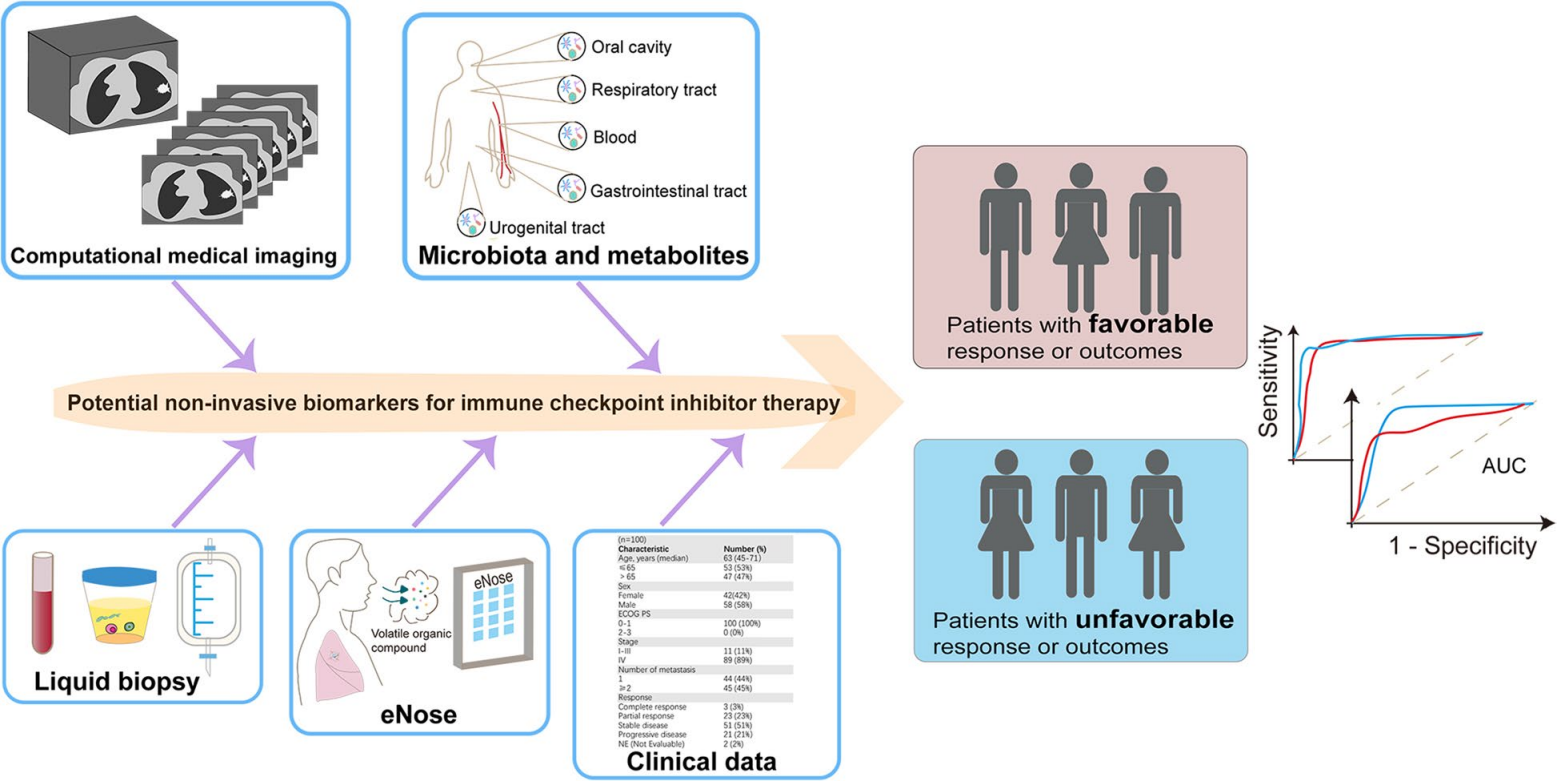
Clinical application of non-invasive biomarkers for ICI efficacy prediction. eNose, Electronic Nose

To date, with the tremendous advances made by interdisciplinary development, a considerable number of non-invasive predictive biomarkers for ICI response have emerged. Here, we provide a relatively comprehensive description of the biomarkers reported during the last seven years, which show significant predictive value and are most likely to be widely used in clinical practice (Table 1). We mainly focus on the recent and salient non-invasive biomarkers based on the radiomic features of medical images, liquid biopsy (LB), microbiota and microbial metabolites, and other biomarkers related to clinical characteristics (Fig. 1), with the aim to provide useful information for the identification of patients who may derive the most benefit from immunotherapy in the future.

**Table 1:** Summary of studies on non-invasive biomarkers for tumor ICI therapy efficacy recently

Type of marker	Marker	Cancer type	Timepoint	Object		Cases	Main findings		Efficacy of marker		Refs
Radiomic-based biomarker	18F-FDG PET/CT signature	NSCLC	Pre-treatment	Patients		99,47,48	18F-FDG PET/CT signatures pre-treatment identified patients benefiting from ICIs		AUC = 0.86, 0.83, 0.81		[21]
	PD-L1 DLS	NSCLC	Pre-treatment	Patients		697	Combined with clinical data, DLS was capable of accurately predicting DCB, PFS, and OS in different cohorts		C-index = 0.70–0.87		[22]
	rADC	Glioblastoma	Post-treatment	Patients		44	Patients with rADC ≥ 1.63 showed longer OS		HR = 0.41, *P* = 0.02		[23]
	Radiomic score of tumor-infiltrating CD8 + T cells	Advanced solid tumors	Pre-treatment	Patients		137	Higher radiomic scores at baseline correlated to a higher proportion of patients with objective response or SD at 6 months and longer OS		*P* = 0.025, 0.013 for objective response and SD respectively HR = 0.58, *P* = 0.0081 for OS		[24]
	Maximum ^89^Zr-labeled CD4 ratio (tumor to heart)	7 different tumor models	Pre-treatment	Mice		35	The ^89^Zr-labeled CD4 ratio > 9 was associated with longer OS		*P* = 0.0018		[25]
	^68^ Ga-grazytracer	Colon Cancer	12 days post-tumor inoculation	Mice		12	The high ^68^ Ga-grazytracer uptake group showed smaller tumor volumes compared with the low uptake group		*P* < 0.05		[26]
	SUVmax of ^89^Zr-labeled atezolizumab	Bladder cancer, NSCLC, and TNBC	Pre-treatment	Patients		22	Patients with CR had a higher SUVmax compared to those with progressive disease The geometric mean SUVmax correlated to PFS and OS		*P* = 0.00021 HR = 11.7, *P* = 0.000028 for PFS; HR = 6.3, *P* = 0.0027 for OS		[27]
	^89^Zr-labeled pembrolizumab	Advanced melanoma or NSCLC	Pre-treatment	Patients		18	The tumor SUVmax was associated with ICI response, PFS, and OS		P trend = 0.014 *P* = 0.0025 for PFS *P* = 0.026 for OS		[28]
Blood-based biomarker	CTCs	NSCLC	Pre- and 4 weeks post-treatment	Patients		104	The presence of CTCs independently predicted the lack of durable response to ICIs at baseline and 4 weeks after treatment		OR 0.28, *P* = 0.02 at baseline; OR 0.07, *P* < 0.01 at four weeks after treatment		[29]
	CTC heterogeneity	Metastatic genitourinary cancer	Pre- and on-treatment	Patients		81	The B and D subtypes were associated with shorter OS at baseline and on C2D1.baseline Increasing CTC heterogeneity correlated to worse OS during the treatment		*P* < 0.0001–0.013 *P* = 0.045		[30]
	PD-L1 expression on CTCs	Metastatic melanoma	Pre-treatment	Patients		25	Patients with PD-L1 + CTCs had longer PFS PD-L1 + CTCs were independent predictors of PFS		PFS, 26.6 vs. 5.5 months, *P* = 0.018 HR = 0.229, *P* = 0.026		[31]
	PD-L1 expression on CTCs	NSCLC	8 weeks post-treatment	Patients		45	Patients with PD-L1 positivity rates ≥ 7.7% at week 8 had longer PFS	*P* < 0.01		[32]	
	Ki67 level of circulating PD-1 + CD8 + T cells	Melanoma	Pre- and 6 weeks post-treatment	Patients		29	Higher Ki67 levels of circulating PD-1 + CD8 + T cells at baseline showed worse OS Patients with the ratio (PD-1 + Ki67 + CD8 + T cell to tumor burden) > 1.94 at 6 weeks post-treatment showed better outcomes in overall response rate, PFS, and OS	*P* = 0.02 *P* < 0.05		[33]	
	TCR diversity and clonality of PD1 + CD8 + T cells	NSCLC	Pre- and post-treatment	Patients		25, 15	Patients with higher TCR diversity pre-ICI had better responses and longer PFS in the combined dataset Patients with increased TCR clonality post-ICI had longer PFS and OS	The optimal Youden’s index = 0.81, Sensitivity = 0.87, Specificity = 0.94 PFS, HR = 0.28; 95% CI 0.11–0.74, *P* = 0.002 OS, HR = 0.23, 95% CI 0.07–0.79; *P* = 0.034		[34]	
	TMR	NSCLC	Pre- and post-treatment	Patients		34	TMR could distinguish responders and non-responders Patients with TMR > 0.39 had longer PFS	AUC = 87% Median PFS, 103 vs. 35 days, *P* = 0.0079		[35]	
	LIPS	Multiple recurrent or metastatic cancer types	Pre-treatment and after the first application	Patients		56, 33	The signature predicted OS benefit accurately The low-risk group had longer OS in the training and validation cohort	C index 0.74 vs. 0.71 Training cohort, HR = 0.26, 95% CI 0.12–0.56, *P* = 0.00025; Validation cohort, HR = 0.30, 95% CI 0.10–0.91, *P* = 0.024		[36]	
	ctDNA	NSCLC, Melanoma, Colorectal Cancer	8 weeks post-treatment	Patients		15	Detection of ctDNA at week 8 correlated with shorter PFS and OS	Median PFS, 11 vs. 2 months, HR 10.2, *P* = 0.001 OS, HR = 15, *P* = 0.004		[37]	
	bTMB	NSCLC	Pre-treatment	Patients		152	The bTMB-high group reached higher ORR values and longer OS	ORR, 35.7% vs. 5.5%, *P* < 0.0001 OS, 23.9 vs. 13.4 months, HR = 0.66, *P* = 0.18		[38]	
	bTMB	NSCLC	Pre-treatment	Patients		50	bTMB levels ≥ 6 was associated with better PFS and ORR	PFS, HR = 0.39, *P* = 0.01 ORR, 39.3% vs. 9.1%, *P* = 0.02		[39]	
	GIN	18 cancer types	6 weeks post-treatment	Patients		44	GIN of cfDNA depicted the ICI efficacy at week 6	HR (NRs vs. Rs) = 5.74, *P* = 0.001		[40]	
	Specific open regions of chromatin	Gastric cancer	Pre-treatment	Patients		32, 52	Patients with high chromatin openness tended to respond to ICIs and had better prognoses	Discovery cohort, Sensitivity 100.0%, Specificity 90.9%, P < 0.001 Validation cohort, Sensitivity 88.9%, Specificity 58.8%, *P* < 0.001 AUC = 0.717		[41]	
	Lung dynamics index	NSCLC	Pre- and within 4 weeks post-treatment	Patients		22	The index differentiated patients with DCB from NDB and correlated with PFS	AUC = 0.93 PFS, HR = 11.38, Wald *P* = 0.006		[42]	
	LIF	Multiple unresectable or metastatic cancer types	Pre-treatment	Patients		95, 292	The LIF-low group had longer PFS, OS, and DCB	Median PFS, 7.4 vs. 1.7 months, 95% CI 2.9–11.9 vs. 1.3–2.1 months, *P* < 0.0001 Median OS, 21.7 vs. 4.3 months, 95% CI 12‒31.4 vs. 3.4–5.1 months, *P* < 0.0001 DCB, 41.7% vs. 6.4%, *P* < 0.0001 AUC = 0.622		[43]	
	HIC	NSCLC	Pre-treatment	Patients		284, 877	The HIC-H group had longer OS in all ICI regimens and ICI monotherapy	Median OS, not-reached vs. 5.0 months, HR = 0.38, *P* < 0.0001 for all ICI regimens OS, 16.8 vs. 2.8 months, HR = 0.36, *P* < 0.0001 for ICI monotherapy		[44]	
	CRAFITY score	HCC	Pre-treatment	Patients	190, 102		Patients with a low CRAFITY score had the longest OS and best radiological responses	*P* < 0.001, C index = 0.62		[45]	
	Circulating exosomal PD-L1	Melanoma	Pre- and 3–6 weeks post-treatment	Patients	39		High levels of circulating exosomal PD-L1 pre-treatment were associated with poor clinical outcomes Responders showed increased exosomal PD-L1 levels at week 3–6 Patients with the fold change value > 2.43 at week 3–6 had better prognoses	*P* = 0.0018 *P* = 0.00001 *P* < 0.05		[46]	
	Circulating exosomal CD73	Melanoma	4 weeks post-treatment	Patients	41		Circulating exosomal CD73 greatly increased in non-responders at week 4 compared with baseline	*P* = 0.0041		[47]	
	EV-score	Gastric cancer	Pre- and at the first month post-treatment	Patients	112		Baseline EV-score could characterize 6-month PD or death EV-score changes at the first month after treatment could predict prognosis	AUC = 0.729, 0.630 PFS, HR = 0.3677, *P* = 0.0471 OS, HR = 0.4568, *P* = 0.1828		[48]	
Microbial biomarker	Microbiota composition	Cutaneous melanoma	Pre-treatment	Patients	94, 5 microbiome datasets		Baseline microbiota composition correlated to the outcome one year after ICI initiation in a cohort of 94 patients Optimized algorithms predicted outcomes across five cohorts consistently	*P* = 0.006 AUC = 0.54–1.00		[49]	
	SCFA	Metastatic or advanced solid tumors	Pre-treatment	Patients	52		Responders had higher levels of fecal and serum SCFAs	*P* < 0.05		[50]	
	SCFA	Multiple myeloma	Pre-treatment	Patients	85		Lower baseline levels of butyrate and propionate were associated with longer PFS	*P* = 0.0015; *P* = 0.0029		[51]	
Exhaled breath	Molecular profiles	NSCLC	Pre-treatment	Patients	92, 51		Baseline data significantly differentiated different responses at 3 months	AUC = 0.89, 0.85		[52]	
	SpiroNose exhaled breath data	NSCLC	6 weeks post-treatment	Patients	62, 32		The eNose was capable of distinguishing objective responders in the early stage	Training, AUC = 0.95, Sensitivity = 100%, Specificity = 73%Validation, AUC = 0.97		[53]	
Other characteristics	Gender	Advanced or metastatic tumors	-	Patients	11,351		Men and women had different ICI outcomes	*P* = 0.0019		[54]	
	BMI	Melanoma	-	Patients	207, 331		Obese patients had improved PFS and OS in the immunotherapy cohort	HR = 0.75, 0.64		[55]	
	BMI	Melanoma	-	Patients	423		To observe the association between BMI and survival outcomes	NS		[56]	
	Body composition	Melanoma	-	Patients	287		Patients featured with sarcopenic obesity showed inferior PFS and those featured with high total adipose tissue index had shorter PFS	HR = 1.4, *P* = 0.04; HR = 1.7, *P* = 0.04		[57]	

*Abbreviations FDG* Fluorodeoxyglucose, *PET* Positron emission tomography, *CT* Computed tomography, *NSCLC* Non-small cell lung cancer, *ICI* Immune checkpoint inhibitor, *AUC* Area under the receiver operating characteristic curve, *PD-L1* Programmed cell death-ligand 1, *DLS* Deeply learned score, *DCB* Durable clinical benefit, *PFS* Progression-free survival, *OS* Overall survival, *rADC* relative Apparent diffusion coefficient, *HR* Hazard ratio, *SD* Stable disease, *89Zr* Zirconium-89, *68 Ga* Gallium-68, *SUVmax* Maximum Standardized uptake values, *TNBC* Triple-negative breast cancer, *CR* Complete remission, *PFS* Progression-free survival, *CTCs* Circulating tumor cells, *OR* Odds ratio, *C2D1* Cycle 2 Day 1, *TCR* T cell receptor, *CI* Confidence interval, *TMR* Ratio of Tregs to Lox-1 + PMN-MDSCs, *LIPS* Signature of the liquid immune profile, *ctDNA* circulating tumor DNA, *bTMB* blood-based Tumor mutation burden, *ORR* Objective response rate, *GIN* Genomic instability number, *NRs* Non-responders, *Rs* Responders, *cfDNA* cell-free DNA, *LIF* Leukemia inhibitory factor, *HIC* Host immune classifier, *CRAFITY* CRP and AFP score in immunotherapy, *HCC* Hepatocellular carcinoma, *EV* Extracellular vesicle, *eNose* Electronic Nose, *SCFA* Short-chain fatty acids, *BMI* Body mass index, *NS* No significance

## Medical image-based radiomic biomarkers

Combined with artificial intelligence (AI), radiomics can capture tumor heterogeneity and provide a complete view of TME phenotypes by quantitatively analyzing medical image features (e.g., texture, region of interest) [58–60]. Owing to their ability to non-invasively and dynamically monitor disease evolution and stratify cancer patients with diverse clinical outcomes, radiomic biomarkers show broad prospects in clinical decision-making [61]. In this part, we will discuss the role of radiomic image signatures, radiotracers, and clinical features in exploring non-invasive radiomic biomarkers for ICI therapy response and prognosis.

### AI and radiomic image signatures

Nowadays, imaging examinations including the combination of computed tomography (CT) and positron emission tomography (PET), and magnetic resonance imaging (MRI) have been used extensively for disease diagnosis and monitoring in routine clinical practice; they may potentially serve as non-invasive tools for developing biomarkers for ICI therapy.

#### Radiomic biomarkers developed by PET/CT

Mu et al. found that the combination of AI and image signatures based on ^18^F-fluorodeoxyglucose (FDG) PET/CT before ICI initiation can predict PD-L1 expression and identify patients with non-small cell lung cancer (NSCLC) who may have a favorable response to treatment [21]. The areas under the receiver operating characteristic curves (AUCs) were 0.86 in training cohorts (*n* = 99, 95% confidence interval [CI] 0.79–0.94), 0.83 in retrospective training cohorts (*n* = 47, 95% CI 0.71–0.94), and 0.81 in prospective test cohorts (*n* = 48, 95%CI 0.68–0.92) respectively. Subsequently, in advanced NSCLC, the authors developed a deeply learned score (DLS) utilizing the the image signatures of 697 patients who received ICI administration and ^18^F-FDG PET imaging [22]. They showed that their PD-L1 DLS differentiated PD-L1 positive and PD-L1 negative patients noticeably (AUC ≥ 0.82). Unexpectedly, DLS performed equally to immunohistochemistry-derived PD-L1 for progression-free survival (PFS) and overall survival (OS). Furthermore, when integrating DLS and clinical data, the score accurately predicted PFS, durable clinical benefit (DCB), and OS in retrospective and prospective testings, and validation cohorts (C-index 0.70–0.87), suggesting that DLS can be a surrogate for immunohistochemistry-based PD-L1 detection to non-invasively guide immunotherapy decisions.

#### Radiomic biomarkers developed using MRI

MRI may outperform PET/CT in brain tumor monitoring. For example, due to the aggressiveness and unique immune environment of glioblastoma (GBM), selection of the optimal therapy for patients with GBM and prediction of treatment efficacy in an early stage are challenging and crucial [62]. Hagiwara et al. dynamically monitored diffusion MRI and investigated the correlation of the data with ICI efficacy in 44 patients with relapsed isocitrate dehydrogenase wild-type GBM [23]. The result showed that the higher the post-treatment relative apparent diffusion coefficient (rADC ≥ 1.63) was, the longer OS (median, 10.3 months vs. 6.1 months) would be (hazard ratio [HR] 0.41; *P* = 0.02), whereas pre-treatment rADC, rADC changes on-treatment, as well as tumor volume did not show an association with OS. Furthermore, Cox regression analysis indicated the correlation between post-ICI rADC and and survival (*P* = 0.02). The possible explanation may be that ADC is negatively correlated with tumor cell density. Therefore, higher post-treatment intra-tumoral ADC predicts better survival, highlighting that diffusion MRI may be a suitable choice for non-invasive prediction of OS benefits in ICI-treated patients with GBM.

#### Radiomic biomarkers related to tumor-infiltrating lymphocytes (TILs)

Studies suggest that the enrichment of TILs is related to favorable outcomes in real-world ICI-treated cohorts [63]. Researchers explored and independently validated a radiomic biomarker for CD8 + TILs to estimate the anti-PD-L1 monotherapy efficacy in a phase 1 trial in multiple solid tumors [24]. Including 8 variables, this marker was validated with the gene expression signature in CD8 + T cells (AUC 0.67; *P* = 0.0019; 95% CI 0.57–0.77) in The Cancer Genome Atlas. The marker could significantly distinguish inflamed (hot) tumors from those immune-desert (cold) tumors (AUC 0.76; 95% CI 0.66–0.86; *P *< 0.0001). Higher radiomic scores at baseline were correlated with longer OS (*P* = 0.0081; HR 0.58; median, 24.3 vs. 11.5 months) and higher proportions of patients with stable disease or objective response (*P* = 0.013 and *P* = 0.025, respectively) at 6 months. Subsequently, the predictive performance of the radiomic signature was verified in another mixed dataset consisting of six independent studies on the combination of radiotherapy and ICI therapy [64].

### AI and radiotracer-related signatures

**Fig. 2:**
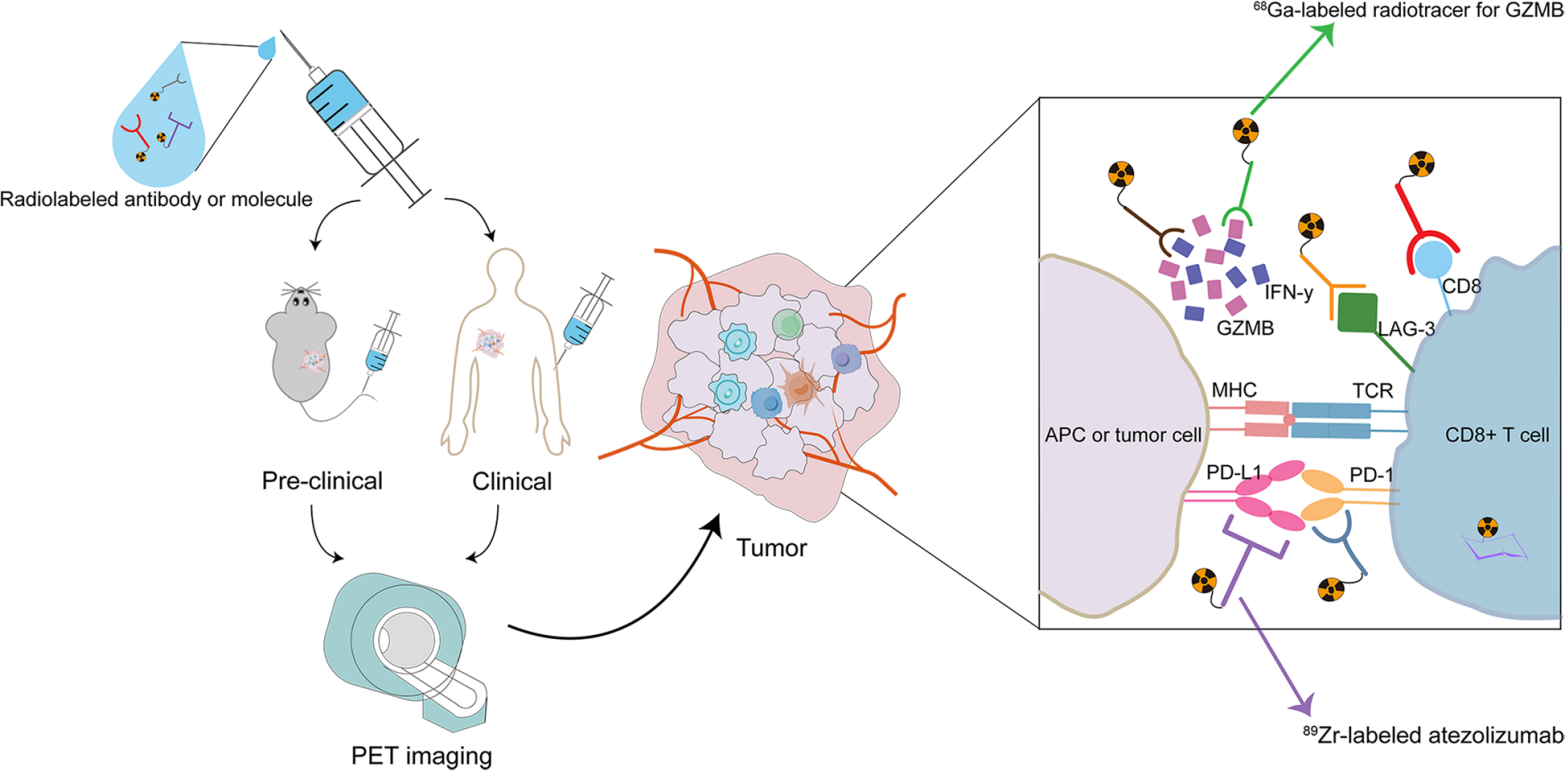
Visualization of tumor and TME with radiotracers and PET imaging in vivo. PET, Positron emission tomography; GZMB, Granzyme B; IFN-γ, Interferon-gamma; MHC, Major histocompatibility complex; PD-L1, Programmed cell death-ligand 1; PD-1, Programmed cell death-1; APC, Antigen presenting cell; LAG, Lymphocyte-activation gene; TCR, T cell receptor; TME, Tumor microenvironment; 89Zr, Zirconium-89; 68 Ga, Gallium-68

The results discussed above demonstrate the potential of combining AI (more specifically, machine learning) with imaging parameters during ICI treatment to develop predictive biomarkers. Additionally, radiotracers may allow a more intuitive visualization of the tumor-intrinsic and TME status, drug distribution, as well as response to ICI at the cellular and molecular levels (Fig. 2).

#### Biomarkers developed with radiotracers at cellular level

By binding to specific molecules on the cell surface, radiotracers can be used to quantify the TIL, which is related to ICI prognosis. In seven types of mouse tumor models (B16F10, 4T1, CT26, MC38, Renca, P815, and Sa1N), Kristensen et al. developed zirconium-89 (^89^Zr)-labeled PET radiotracers for the specific detection and assessment of systemic CD8a + and CD4+ TILs [25]. With high radiochemical purity and immunoreactivity (> 99% and > 85% respectively), the tracers were able to phenotype the tumor model as “hot” or “cold” and predict response to ICI therapy. Mice with the maximum CD4 ratio (^89^Zr-labeled, tumor to heart) > 9 had longer OS (*P* = 0.0018).

#### Biomarkers developed with radiotracers at molecular level

In addition to cellular level tracing, small molecules in TME can also be traced. Heskamp et al. were the first to confirm that non-invasively visualizing the PD-L1 expression in vivo was possible [65]. Using radiolabeled single-domain antibodies in MC38-bearing mice, researchers observed the compensatory upregulation of lymphocyte-activation gene (LAG)-3 on TILs after PD-1 blockade. The cooperative effect of the combination use of anti-PD-1 and anti-LAG-3 delayed tumor growth [66], providing new insights into the failure of anti-PD-1 monotherapy. In addition, granzyme B, secreted by cytotoxic T cells in the TME, can be quantified and specifically targeted by PET imaging probes (i.e., gallium-68 [^68^ Ga]-grazytracer) in vivo [26, 67]. With warranted safety, great stability, and targeting efficiency, the ^68^ Ga-grazytracer excellently predicted the response to ICIs in mice colon cancer models (i.e., the higher uptake group was associated with smaller tumor volumes, *P* < 0.05) [26], exhibiting higher sensitivity than ^18^F-FDG [68]. Moreover, in a clinical trial in multiple tumor models, positive ^68^ Ga-grazytracer PET imaging results were associated with a favorable clinical response [68]. Furthermore, radiotracers can be used to trace the biodistribution and metabolism of drugs. In one study, investigators innovatively used ^89^Zr to label atezolizumab in 22 patients across three different tumor types and recorded the PET signals [27]. They concluded that compared to patients with instant progressive disease, those with complete remission had higher maximum standardized uptake values (SUVmax) (*P* = 0.00021). The geometric mean SUVmax was also correlated with PFS (HR 11.7; 95% CI 3.3–62.7; *P* = 0.000028), and OS (HR 6.3; 95% CI 1.8–33.4; *P* = 0.0027). Similarly, in their recent study on advanced melanoma or NSCLC, the researchers used ^89^Zr to label pembrolizumab in patients and confirmed the association between tumor SUVmax and therapeutic response (*P* trend = 0.014), OS (*P* = 0.026), and PFS (*P* = 0.0025) [28].

To summarize, the combination of AI, radiomic image features, radiotracers, and clinical features may collectively contribute to the development of predictive biomarkers for ICI efficacy; however, they are subject to certain limitations. First, patients may be allergic or intolerant to contrast agents. Second, some analyses are retrospective and lack adequate samples. Therefore, these results are required to be validated in larger prospective datasets. Third, the timing of imaging varies among studies, protocols for the extraction and processing of image features need to be standardized, and algorithms need to be optimized. Nevertheless, as imaging is extensively utilized in conventional clinical practice and can repeatedly evaluate and monitor the phenotypic changes in a completely non-invasive and innovative manner, radiomics still offers promising prospects in ICI efficacy prediction.

### Liquid biopsy

**Fig. 3:**
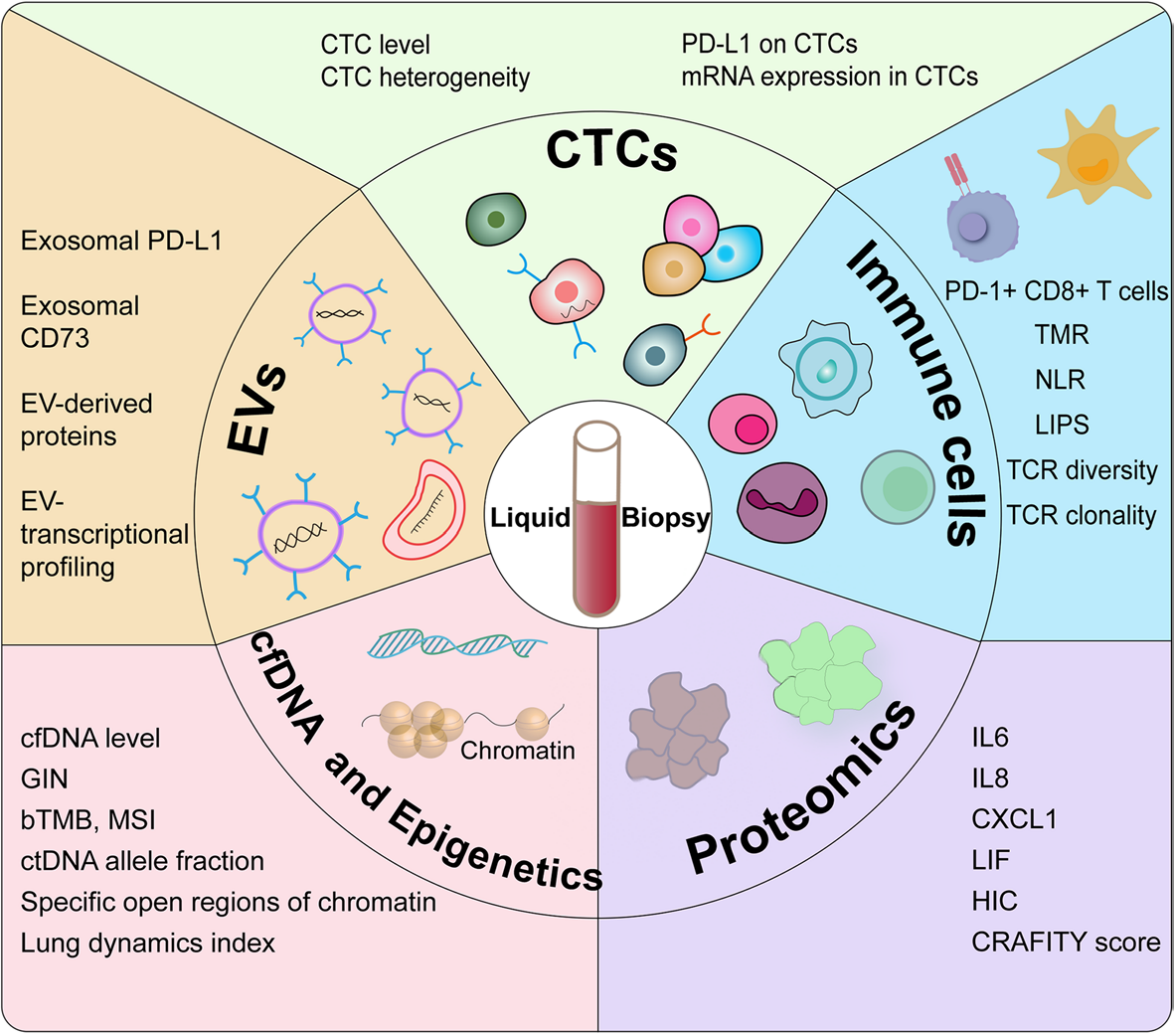
Main blood-based biomarkers for ICI efficacy prediction. CTC, Circulating tumor cell; PD-L1, Programmed cell death-ligand 1; PD-1, Programmed cell death-1; TMR, Ratio of Tregs to Lox-1 + PMN- myeloid-derived suppressor cells; NLR, Neutrophil-to-lymphocyte ratio; LIPS, Signature of the liquid immune profile; TCR, T cell receptor; IL, Interleukin; CXCL, C-X-C motif chemokine ligand; LIF, Leukemia inhibitory factor; HIC, Host immune classifier; CRAFITY, CRP and AFP in immunotherapy; cfDNA, cell-free DNA; GIN, Genomic instability number; bTMB, Blood-based tumor mutation burden; MSI, Microsatellite instability; ctDNA, circulating tumor DNA; EV, Extracellular vesicle

In general, LB refers to relatively non-invasive biological detection from bodily fluids, mostly blood, but also urine, pleural effusion, saliva, cerebrospinal fluid, and others [69]. Compared with tissue biopsy, LB is minimally invasive and can overcome tumor heterogeneity, better reflecting the overall landscape of the tumor and TME [70]. Owing to its extensive availability and low cost of sampling, dynamical monitoring of tumor evolution and timely prediction of response to ICIs are possible and allow treatment plan modification. Traditionally, LB, mainly focusing on tumor characteristics, includes circulating tumor cells (CTCs), proteins and extracellular vesicles, and circulating cell-free DNA or tumor DNA more specifically (cfDNA or ctDNA) (Fig. 3). Moreover, the roles of circulating immune cells and related characteristics (which are usually not considered to be parts of LB) in mirroring host immune status are equally non-negligible [71].

### CTCs

#### CTC quantity and heterogeneity

CTCs refer to single tumor cells or cell clusters that are shed into peripheral blood circulation from tumor lesions (primary or metastatic) [72] and are usually regarded as metastatic-related precursor cells [73]. Evidence has shown that CTC quantity and heterogeneity can serve as potential indicators of ICI efficacy. For instance, in an investigation of 104 patients with advanced NSCLC, the presence of CTCs was demonstrated to independently predict the lack of durable response both before ICI initiation (*P* = 0.02, odds ratio 0.28) and 4 weeks after ICI treatment (*P* < 0.01, odds ratio 0.07), and was also associated with poor PFS and OS [29]. In addition, Chalfin et al. detected CTCs in peripheral blood before and during combined immunotherapy in metastatic genitourinary cancer (*n* = 81) [30], and they found that two CTC subtypes with specific cellular features were linked with inferior OS before treatment and on cycle 2 day 1 (0.9–2.3 months vs. 28.2 months; *P* < 0.0001–0.013). Moreover, a trend toward worse OS as CTC heterogeneity increased during the treatment was observed (from baseline to cycle 2 day 1, *P* = 0.045). The researchers speculated that the underlying mechanism may be that the differences in CTC morphology are the result of tumor mutation burden (TMB). Nevertheless, validation in other cancer types and larger independent cohorts is necessary to confirm its predictive value.

#### PD-L1 expression on CTCs

Evidence has shown that PD-L1 expression on peripheral CTCs correlates with that in tumor tissues [74], suggesting its potential capacity to predict ICI efficacy as a surrogate marker. Investigators identified that PD-L1 on CTCs not only assisted in distinguishing responders (Rs), non-responders (NRs), or pseudoprogressors in the early stage of ICI administration, but also had an association with prognosis in advanced melanoma [31]. Patients with PD-L1 + CTCs showed longer PFS in comparison with those whose CTCs had no PD-L1 expression (26.6 vs. 5.5 months; *P* = 0.018). Multivariate linear regression analysis further substantiated the capacity of PD-L1 + CTCs to predict PFS independently (HR 0.229; 95% CI 0.052–1.012; *P* = 0.026). Another study conducted a longitudinal tracking of 45 patients with NSCLC and evaluated the PD-L1 expression on CTCs during nivolumab administration and found that, at week 8, patients whose PD-L1 positivity rates were ≥ 7.7% had significantly longer PFS (*P* < 0.01) [32]. However, it was identified as a negative prognostic predictor for ICI treatment in other studies owing to an excessively high proportion of CTCs expressing PD-L1 and inconsistent sampling time [75], hindering the assessment of its predictive value.

CTCs are usually very rare, with only 1–100 cells detected in 1 ml blood and a quite short half-life of 1–2.4 h in circulation [76], and diverse methods may enrich various CTC subtypes; therefore, the detection of CTCs remains technically challenging [71]. Recently, a new technique has been used to detect mRNA expression in captured CTCs, indicating that the transcriptional profiles can provide valuable information on prognostic prediction in metastatic prostate cancer; however, CTC quantity and leukocyte lysis may influence test results [77]. Therefore, although biomarkers based on CTCs and related features offer crucial information for ICI efficacy prediction, the isolation and detection methods need to be standardized, and larger-scale prospective validation is required.

### Circulating immune cell-related biomarkers

#### Circulating immune cells with anti-tumor function

Intratumoral immune signatures can serve as independent prognostic factors in cancer [78]. Moreover, the systemic immune status plays a vital role in ICI therapy and can also be influenced by tumor burden; accordingly, biomarkers of systemic immunity may help guide treatment decisions [79]. Owing to the minimal invasiveness and real-time monitoring of the systemic immune status using blood samples, many investigations have recently focused on biomarkers based on circulating immune cells (e.g., monocytes, the ratio of neutrophil-to-lymphocyte or eosinophil) [80–82], among which, T cells have attracted most attention on account of their indispensable contributions to tumor immunity. Notably, PD-1 + CD8 + T cells are representative of a subtype of "exhausted" T cells (T_ex_ cells). In advanced melanoma, Huang et al. demonstrated that patients with higher Ki67 levels of PD-1 + CD8 + T cells in the circulation before immunotherapy displayed worse OS (*P* = 0.02) than those with lower Ki67 levels [33]. It is speculated that high Ki67 levels in that subset of T cells pre-ICI treatment reflect the active proliferation and metabolic status and obvious immune responses, which are correlated with high tumor burden in the host; therefore, they have been proposed as poor prognostic indicators. In contrast, after being targeted by the anti-PD-1 monotherapy, the T_ex_ subpopulation gains the ability to reactivate, and their early proliferation has been identified as associated with positive clinical outcomes of ICI therapy [83]. Interestingly, T_ex_ cells expanded in patients with favorable responses, while effector CD8 + T cells were enriched in those with progressive disease, confirming the significance of T_ex_ cell reinvigoration in responding to ICIs [33]. However, if the tumor burden is too high, achieving efficient therapeutic outcomes would be difficult even after the robust reinvigoration with ICIs. Therefore, the imbalance between tumor burden and T_ex_ cell reactivation results in a clinical ICI treatment failure in most cases rather than the inability to induce immune reinvigoration. Therefore, considering both factors may be more rational in ICI efficacy prediction. Researchers determined a ratio (Ki67 + PD-1 + CD8 + T cell to tumor burden) of 1.94 by classification and regression tree analysis [33], which turned out to strongly differentiate patients with different clinical outcomes as early as week 6 post-treatment; the ratio > 1.94 was correlated with better outcomes in overall response rate, PFS, and OS (*P* < 0.05). Overall, the above evidence demonstrates the dual indicative role of T_ex_ cells in ICI efficacy prediction.

#### T cell receptor diversity and clonality

Receptor diversity of T cells (TCR) can reflect their ability to recognize neoantigens. As discussed above, the PD-1 phenotype represents the exhausted state of CD8 + T cells; therefore, this subtype may include neoantigen-specific cytotoxic T lymphocytes. In addition, PD-1 + CD8 + T cells in circulation and the TME are similar in TCR repertoires [84]. Hence, the TCR repertoires of peripheral PD-1 + CD8 + T cells may function as substitutes for those in the tumor and provide predictive information during ICI administration. In one study, Han et al. focused on PD-1+CD8+ T cells in patients with NSCLC, sequenced the complementarity determining region 3 of TCRβ chains and explored its predictive value in ICI therapy [34]. They demonstrated that in contrast to those with low diversity, patients with high TCR diversity pre-ICI exhibited better responses and longer PFS (6.4 vs. 2.5 months; *P* = 0.021) in a dataset (*n* = 25; HR 0.39; 95% CI 0.17–0.94), which was verified in another dataset (*n* = 15). In the combined cohorts, the optimal Youden’s index was 0.81 (with a specificity of 0.94 and a sensitivity of 0.87). Moreover, patients whose TCR clonality of PD-1 + CD8 + T cells increased post-ICI showed superior PFS and OS (7.3 vs. 2.6 months and not reached vs. 7.5 months; *P* = 0.002 and 0.034; HR 0.28 and 0.23; 95% CI 0.11–0.74 and 0.07–0.79, respectively) than those whose clonality decreased [34]. Hence, their results highlight that in NSCLC, the clonality and diversity of TCR from circulating PD-1 + CD8 + T cells are promising non-invasive predictive indicators of response to ICIs and survival prognosis in NSCLC, which was supported by a subsequent study [85].

#### Circulating immune cells with immunosuppressive effects

In addition to the anti-tumor subpopulation, some immunosuppressive components in the TME, which promote tumorigenesis and progression [86], can also provide useful information for predicting immunotherapeutic responsiveness [87, 88]. In a cohort of patients with NSCLC (*n* = 34) on anti-PD-1 monotherapy, Kim et al. analyzed regulatory T cells (Tregs) and myeloid-derived suppressor cells (MDSCs) in peripheral blood [35] and found that compared with the frequency of either cell type alone, the difference in the TMR (Tregs to Lox-1 + PMN- MDSCs ratio) between Rs and NRs was greater (AUC 0.87) and PFS (*P* = 0.0079; median, 103 vs. 35 days) was considerably longer in patients whose TMRs were > 0.39. These findings were confirmed in another validation cohort (*n* = 29), indicating the unignorable role of immunosuppressive cells in ICI outcomes prediction in NSCLC.

#### Comprehensive assessment of circulating immune cells

Compared to the individual evaluation of circulating anti-tumor or immunosuppressive cells, a combined evaluation may help identify biomarkers with more robust predictive performance. A prospective analysis of the immune status in patients treated with ICI across different recurrent or metastatic cancer types identified a LIPS, which is a signature of the liquid immune profile developed based on five subtypes of immune cells (specific subtypes of monocytes, T cells, neutrophils, natural killer T cells and dendritic cells) [36]. In their analysis, the signature reached a high level of accuracy in predicting prognostic benefit (C-index 0.74 vs. 0.71), with significantly longer OS in the low-risk cohort in both the training and validation datasets (*n* = 56 and 33, HR 0.26 and 0.30; 95% CI 0.12–0.56 and 0.10–0.91; *P* = 0.00025 and 0.024, respectively). LIPS also predicted the PFS in the combined cohort. In addition, after the first course of ICI, two types of LIPS (neutrophils and natural killer T cells) can indicate survival outcomes (PFS and OS) dynamically. Overall, the identified LIPS signature is a simple, effective, and low-cost biomarker potential to serve as a predictor for the prognosis of cancer patients undergoing ICI therapy.

### cfDNA-associated biomarkers

#### cfDNA and ctDNA quantification

cfDNA, first identified by Mandel and Metais in 1984 [89], refers to a mixture of nucleic acids released through cell secretion, necrosis, or apoptosis into the bloodstream [90], including ctDNA [91]. ctDNA carries tumor-specific features and the genetic and epigenetic variation has been an appealing alternative in cancer diagnosis and prognosis prediction [92, 93].

In recent years, several studies have reported using cfDNA or ctDNA can predict ICI response and prognosis. A prospective study of anti-PD-1 therapy across three types of cancer (NSCLC, melanoma, colorectal cancer) identified a notable relationship between the synchronous changes of tumor size and ctDNA levels at week 8 after treatment (*r* = 0.86; *P* = 0.002) [37], which was corroborated by subsequent findings [94]. Furthermore, the detection of ctDNA at week 8 also correlated with shorter PFS (median, 11 vs. 2 months; HR 10.2; 95% CI 2.5–41; *P* = 0.001) and OS (HR 15, *P* = 0.004) [37].

#### cfDNA mutation-based biomarkers

##### Blood-based TMB

In addition to quantification of cfDNA level, mutation characteristics of the genome may also help predict the response to ICIs. Currently, tissue-based TMB (tTMB) is commonly used to predict immunotherapy efficiency in clinical practice; nevertheless, it is subject to heterogeneity, interference by other factors [95], and potential risks of metastasis induced by invasive detection. Owing to the close correlation between blood-based TMB (bTMB) and tTMB [96, 39], an abundance of investigations have focused on the application of bTMB in ICI efficacy prediction.

A retrospective assessment of two randomized controlled trials confirmed the positive relationship between tTMB and bTMB (Spearman’s rank correlation, r = 0.64, 95% CI 0.56–0.71) and revealed that bTMB could identify patients with NSCLC sensitive to atezolizumab with good repeatability and predict PFS independently, regardless of PD-L1 expression levels [96]. Furthermore, utilizing the bTMB cutoff score defined in this study [96], in patients with NSCLC on first-line atezolizumab treatment (n = 152), Kim et al. prospectively assessed the relationship between bTMB and clinical outcomes [38]. They observed that patients with bTMB ≥ 16 (bTMB-high group) reached a more favorable objective response rate (ORR) (35.7% vs. 5.5%; 95% CI 19.2–55.5 vs. 2.2–12.2; *P* < 0.0001) and longer OS (23.9 vs. 13.4 months; HR 0.66; *P* = 0.18; 90% CI: 0.40–1.10) than those with bTMB < 16. Moreover, the ORR values improved with the increase of the bTMB cutoffs. In addition to assessing bTMB directly, Wang et al. designed a cancer gene panel with an improved gene panel size and algorithm to estimate bTMB in NSCLC [39]. In their study, bTMB levels ≥ 6 were associated with better ORR (39.3%; 95%CI 23.9–56.5% vs. 9.1%, 95%CI 1.6–25.9%; *P* = 0.02) and PFS (HR 0.39; *P* = 0.01; 95%CI 0.18–0.84).

##### Other cfDNA mutation-related biomarkers

In addition to bTMB, other cfDNA- and ctDNA-based genomic biomarkers have also been reported to show excellent predictive performances. Jensen et al. developed the genomic instability number (GIN) [40] to evaluate the copy-number alterations among 18 different types of malignancies and demonstrated that GIN from cfDNA could predict PFS at approximately week 6 after ICI initiation (*n* = 44, HR 5.74; 95% CI 1.9–17.7; *P* = 0.001). Surprisingly, dynamic changes in GIN levels during treatment distinguished ICI responders even before radiomic imaging. As demonstrated by Ricciuti et al., early changes in ctDNA allele fraction were associated with radiomic response and long-term clinical efficacy in NSCLC [94]. Furthermore, microsatellite instability detection based on cfDNA was also available [97], with the higher chromosomal instability group exhibiting better ICI responses in patients with prostate cancer than the lower chromosomal group. Collectively, the findings suggest that cfDNA mutation-related features are of great value in ICI efficacy prediction.

However, cfDNA mutation does not directly provide information on the antigenicity and presentation of tumor-associated neoantigens. Therefore, the biological differences between bTMB and the true quality or quantity of neoantigens can vary among different tumor types. Hence, other factors, such as major histocompatibility complex-1 genotype and loss of heterozygosity for human leukocyte antigen, which potentially affect the immune response, need to be incorporated, and adequate efforts to optimize algorithms will be required in the future [38].

#### cfDNA epigenetic-based biomarkers

Genomic instability and mutation are recognized as fundamental hallmarks of tumorigenesis and pathogenesis. A purely epigenetic regulation of gene expression, known as "non-mutational epigenetic reprogramming" [98], has been demonstrated to be related to the development of cancer [99]. Epigenetic signatures can reveal features beyond genetic mutation and determine the originating tissues of the molecules in peripheral blood [100], with DNA methylation being one of the most concerned.

Investigations have indicated that DNA methylation status in tumor tissues is associated with the prognosis of ICI therapy [101]. In a recent report, using eQTM (expression quantitative trait methylation) analysis based on tumor tissues in melanoma [102], researchers illustrated that three cis-eQTM CpGs were closely associated with the immune cytolytic activity score and could be used as surrogates for it. One eQTM in transcription factor 7 was shown to provide information on the overall status of T cell differentiation and exhaustion; therefore, it can be used as a prognostic biomarker independent of the cytolytic activity score. Owing to the high stability and tissue specificity in bodily fluids [103] and the consistency of methylation pattern between cfDNA and DNA in original cells [104], the exploration of methylation biomarkers from LB is a new area of interest. Research has revealed that ctDNA methylation can be used for early disease screening, tissue origin tracking, and chemotherapy efficiency assessment [105, 106]. In patients with gastric cancer on anti-PD-1 treatment, Shin et al. determined specific open regions of chromatin to distinguish Rs from NRs by quantitatively evaluating the accessibility of genome-wide chromatin of peripheral blood CD8 + T cells at baseline [41]. Encouragingly, when using nine indexes in combination, patients with gastric cancer with high chromatin openness achieved a clear response and had superior PFS (discovery cohort, *n* = 32, sensitivity 100.0%, specificity 90.9%, median, unreached vs. 2.7 months, *P* < 0.001; validation cohort, *n* = 52, sensitivity 88.9%, specificity 58.8%, median, 7.6 vs. 1.6 months, *P* < 0.001; AUC 0.717). Moreover, a recent report integrated machine learning into the sequencing of gene promoters from cfDNA to infer epigenetic expression profiles at the single-gene resolution and developed a “lung dynamics index” [42]. They analyzed 44 blood specimens of 22 patients with NSCLC at baseline and within 4 weeks after PD-L1 blockade initiation, demonstrating that epigenetic signatures can reliably predict ICI prognosis. In their analysis, this epigenetic metric reliably differentiated patients with DCB and no durable clinical benefit (AUC 0.93, 95% CI 0.78–1.00) and had a clear correlation with PFS (HR 11.38, Wald *P* = 0.006). Taken together, the above evidence indicates that cfDNA-related characteristics (including cfDNA quantification, genomics, and epigenetics) can identify patients who may most benefit from ICI therapy.

### Circulating proteomic profiling in plasm or serum

Bridging the gap between genome and phenotype, proteomic signatures in plasma or serum are unique protein patterns that correlate with tumor burden and immune response of patients with cancer. Loriot et al. [43] first performed a large-scale analysis of the plasma proteome of patients with advanced malignancies on ICI therapy and found that compared with interleukin-8, interleukin-6, and C-X-C motif chemokine ligand-1, which have previously been identified to be correlated with ICI outcomes [107, 108], leukemia inhibitory factor (LIF) had the strongest relationship with clinical prognosis and was independent of PD-L1 status or other indicators. In comparison with the LIF-high cohort, the LIF-low cohort showed superior DCB (41.7% vs. 6.4%, *P* < 0.0001), PFS, and OS (median, 7.4 vs. 1.7 months and 21.7 vs. 4.3 months; 95% CI 2.9–11.9 vs. 1.3–2.1 and 12–31.4 vs. 3.4–5.1, for PFS and OS respectively; *P* < 0.0001), which was validated in an independent cohort (*n* = 292, AUC 0.622). Hence, they speculated that LIF plays a critical role in cancer immunotherapy resistance and can be developed as a robust predictor. Furthermore, targeting the LIF axis may provide promising insights into the improvement of treatment efficiency, especially in patients with high plasma LIF levels.

Advanced techniques for protein identification or quantification (e.g., mass spectrometry, affinity-based proteomic assays), in combination with machine learning algorithms provide a promising approach for the identification of predictive proteomic biomarkers for immunotherapy [109]. In a prospectively-designed observational study in NSCLC, the researchers developed a host immune classifier (HIC) based on serum proteomics and evaluated its performance in ICI outcome prediction [44], revealing that HIC can identify patients benefiting from ICI, regardless of combined therapy. Specifically, for patients on all ICI regimens, a significant difference in survival outcomes between the HIC-Hot (HIC-H) and the HIC-Cold (HIC-C) groups was observed (*n* = 196, 88; HIC-H vs. HIC-C: median OS, not-reached vs. 5.0 months, 95%CI 15.4–undefined vs. 2.9–6.4; HR 0.38, 95%CI 0.27–0.53; *P* < 0.0001). In terms of patients treated with ICI monotherapy, OS was 16.8 for HIC-H and 2.8 months for HIC-C (HR 0.36, *P* < 0.0001). Additionally, the prediction efficiency was independent of PD-L1, implicating a better predictive performance if the two factors are combined. Nevertheless, this is an observational and non-randomized study that requires rigorous multi-institutional design and extensive independent cohorts to prospectively validate. However, the current results are of great significance for guiding clinical immunotherapy decisions.

Surprisingly, studies have recently revealed that some plasma proteins routinely detected in clinical practice also show a notable predictive value. For instance, multivariate analysis indicated that C-reactive protein (CRP) and serum alpha-fetoprotein (AFP) at baseline were independent predictors of the prognosis of PD-L1-based therapy in hepatocellular carcinoma (HCC). Based on these findings, Scheiner et al. developed an easily applicable score with CRP and AFP in immunotherapy (CRAFITY) [45]. Patients with a low CRAFITY score had the best radiological responses (highest disease control rate, *P* < 0.001) and the longest OS (27.6 months, 95% CI 19.5–35.8) in the discovery cohort (*n* = 190), followed by CRAFITY-intermediate patients (11.3 months; 95% CI 8.0–14.6), and CRAFITY-high patients (6.4 months; 95% CI 4.8–8.1; *P* < 0.001). The results were verified in another independent external cohort (*n* = 102, C-index 0.62). Indeed, the combination of AFP and CRP in ICI outcome prediction in HCC is rational at the mechanism level; CRP, an acute-phase protein, is a widely-recognized systemic marker of inflammation induced by cancer, and inflammation can contribute to tumorigenesis and disease progression [110, 111]. Recent evidence shows that CRP can promote tumor immunosuppression [112]. In addition, AFP is associated with angiogenesis, hampers anti-tumor immunity [45], and facilitates tumor proliferation [113]. Nonetheless, some investigators disagree, arguing that the findings of this retrospective study are required to be further validated in large clinical studies. Of note, considering that the CRP and AFP levels may be affected by other diseases not related to HCC (e.g., infection), and there is heterogeneity among patients in terms of their liver function status, treatment line, and the specific ICI type, the integration of additional indicators may help overcome selection bias and optimize the prognostic performance.

In contrast to DNA or RNA-based studies, proteomics can explore post-translational modifications and analyze proteins quantitatively and qualitatively, allowing in-depth profiling of the host immune response and TME, as well as the identification of biomarkers for ICI outcomes [109]. However, due to limitations of sample preparation procedures, identification of protein isoforms, and retrospective studies, the applicability of some proteomic biomarkers in clinical practice remains challenging.

### Extracellular vesicles

Extracellular vesicles (EVs), which are secreted by multiple cell types under physiological conditions and stress, can be roughly classified into exosomes, microvesicles, and apoptotic bodies [114]. Consisting of proteins, lipids, and nucleic acids delivered by parental cells to recipient cells, EVs are considered mediators in intercellular communication [115]. Owing to the immunogenicity, molecular delivery functions, and different cellular origins, exosomes play dual roles in tumorigenesis and development in various cancer types [116]. Specifically, immune cell-derived exosomes usually execute potent antitumor activity [117], whereas tumor cell-derived exosomes, possessing similar functions to their parental cells, are linked with distant tumor metastasis and immune escape [118–120]. Accordingly, exosomes may carry meaningful information regarding ICI efficacy prediction.

Chen et al. found that in patients with metastatic melanoma on anti-PD-1 therapy [46], higher levels of baseline circulating exosomal PD-L1 pre-treatment were correlated with poorer clinical outcomes (*P* = 0.0018). Furthermore, at 3–6 weeks after ICI initiation, responders showed a greater elevation of circulating exosomal PD-L1 levels released by metastatic melanoma cells (*P* = 0.00001). A 2.43-fold change identified by ROC analysis stratified patients with different clinical responses, with the value > 2.43 at week 3–6 related to better prognoses (ORR, PFS, and OS, *P* < 0.05). The favorable prediction of increasing PD-L1 may be a result of T cell proliferation and reinvigoration successfully triggered by anti-PD-1 treatment, represented by circulating exosomal PD-L1 levels. In another study of melanoma, consistent changes of PD-L1 were obtained at an early stage of treatment, and in comparison with baseline, circulating exosomal CD73 increased remarkably at week 4 (*P* = 0.0041) in NRs but not in Rs [47]. Recently, Zhang et al. comprehensively assessed the plasma EV-derived protein spectrum in patients with gastric cancer on ICI-based therapies and then developed an “EV-score” [48]. A high “EV-score” reflected a microenvironment with strong anti-tumor immunity features. The baseline EV-score reached high AUCs in predicting 6-month disease progression or death (AUC = 0.729 and AUC = 0.630, respectively). Moreover, the EV-score changes at the first month after ICI initiation predicted the prognosis (HR = 0.3677 and 0.4568, *P* = 0.0471 and 0.1828, for PFS and OS respectively, HR = 0.4568). This EV-score they developed is a stable index for stratification and dynamic prediction of prognosis during immunotherapy. Additionally, at the transcriptional level, EV transcriptional profiling revealed drivers of ICI resistance and melanoma progression, which correlated with clinical response to ICI [121].

**Fig. 4:**
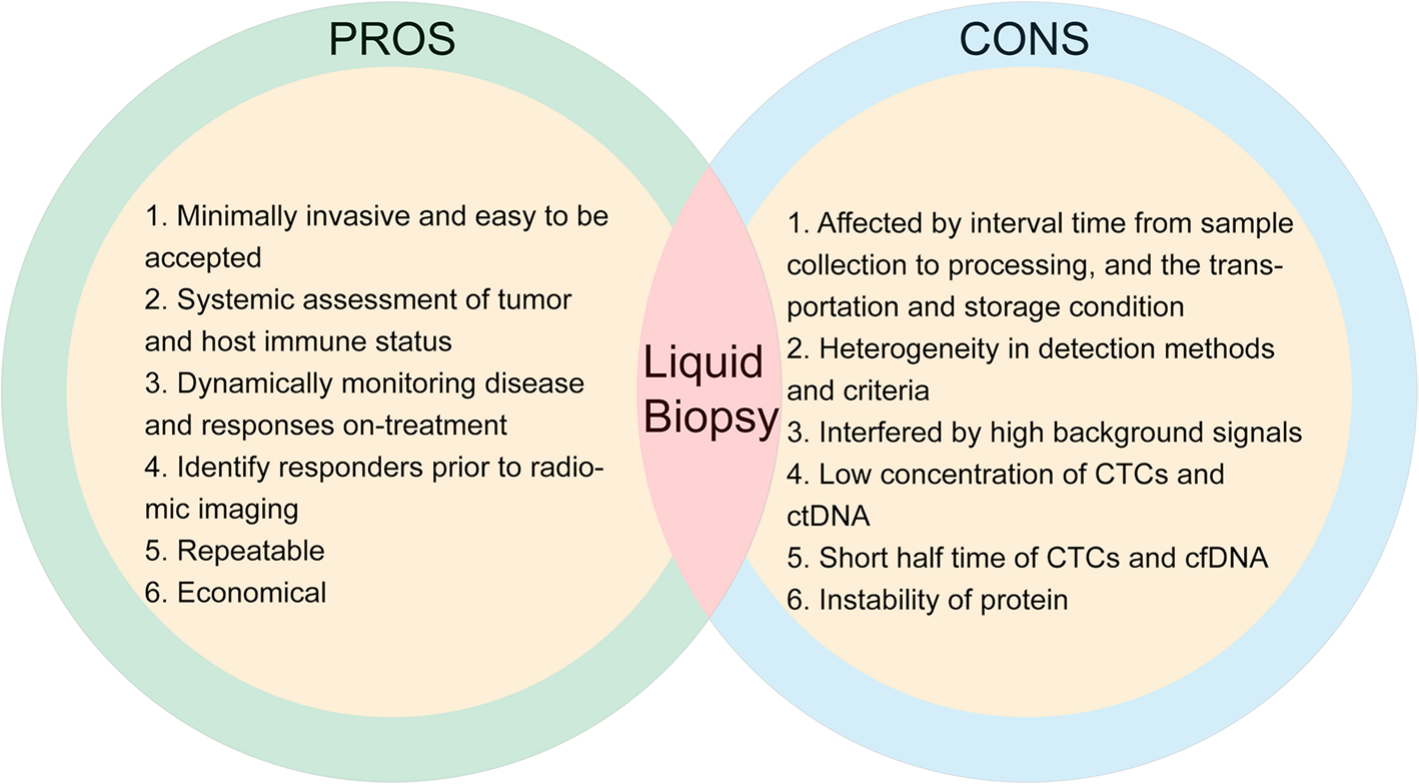
Pros and cons of blood-based biomarkers. CTCs, Circulating tumor cells; ctDNA, circulating tumor DNA; cfDNA, cell-free DNA

In clinical practice, obtaining a sufficient amount of tumor tissues with adequate quality from patients for cellular and molecular testing can be challenging. Owing to its minimally invasive, readily available, reproducible, and relatively low-cost features, LB has become an attractive approach that can provide comprehensive insight into the tumor and systemic immune profiles [70]. Peripheral blood-based biomarkers offer clinicians abundant information for rapid decision-making and dynamic assessment of therapeutic efficacy, showing promise for wider application. Nevertheless, there are some challenges ahead (Fig. 4). First, some circulating biomarkers (e.g., CTC, ctDNA) have comparatively low concentrations and short half-lives in peripheral blood [76]; for example, the half-life of cfDNA varies from 16 min to 13 h [122, 123], which makes it challenging to capture and hinders immediate application in early efficacy evaluation [42]. Second, high background signals from other cells may interfere with the analysis. Third, heterogeneous methods of detection and analysis used at different institutions may lead to inconsistent results, with different cell types enriched and different cutoff points for biomarkers identified. Lastly, factors including the time interval from sampling to processing of specimens and the transport and storage temperature may affect the cellular state and stability of cfDNA and proteins. Consequently, the development of highly-sensitive techniques and formulation of standardized guidelines for pre-analytical procedures of patient specimens are urgently needed [124] to stimulate the identification of more robust biomarkers.

### Microbiota and microbial metabolites

In addition to the medical image-based radiomic biomarkers and markers developed by LB, the microbiome is also a new factor for predicting ICI efficacy. Interestingly, host immunity can modulate the microbiome by altering bacteria-associated signals, and conversely, the microbiome (especially intestinal-derived) can shape the immune system of the host by locally and systemically regulating immune responses [125–127], partly attributed to the stimulation of host pattern recognition receptors by the cross-presentation of tumor and microbial antigens [128]. Surprisingly, ICI Rs-derived fecal transplantation helped patients overcome resistance to ICIs [129], providing convincing evidence that ICI efficacy can be influenced by the host gut microbiome [130].

For the past few years, a considerable number of investigations have discovered specific gut microbes associated with ICI efficacy. However, they used diverse analytical approaches, lacking consistency and reliable reproducibility. For example, by analyzing the baseline fecal microbiome samples of patients with melanoma on ICI treatment, Gopalakrishnan et al. demonstrated the abundance of *Ruminococcaceae* bacteria in Rs (*n* = 43, *P* < 0.01) [131]. In other studies, *Collinsella aerofaciens*, *Bifidobacterium longum*, and *Enterococcus faecium* were abundant in Rs (*n* = 42, *P* = 0.004) [132], and *Bacteroides caccae* was identified in Rs for all types of ICI therapies (*n* = 39, *P* = 0.032) [133]. In contrast, *Ruminococcus gnavus* and *Bacteroides* were shown to be related to shorter PFS in regression analysis of both 16S and shotgun data (*r* = 0.32, *P* = 0.1; *r* = 0.89, *P* < 0.001) [134]. Nevertheless, most studies were based on retrospective analyses and included limited cohorts from a single institution.

To address the issue of inconsistency, McCulloch et al. recently evaluated a new dataset of patients with melanoma on ICI therapy (*n *= 94) [49]. The Time-to-event analysis demonstrated that approximately one year after ICI initiation, baseline microbiota composition was greatly correlated with the outcomes (*P* = 0.006). The unfavorable microbiota mainly consisted of Gram-negative bacteria, which can promote lipopolysaccharide-dominated inflammation in the gut and lead to local and systemic inflammation, ultimately manifested by poor prognosis. Next, the authors integrated bioinformatics into the meta-analysis of five microbiome cohorts of anti-PD-1 therapy in melanoma and reported that the taxa correlated with superior responses mainly were the *Actinobacteria* phylum and two families of *Firmicutes*, while those associated with unfavorable responses were mainly Gram-negative bacteria. Of note, optimized learning algorithms trained with batch-corrected microbiome data estimated ICI outcomes across all cohorts consistently (AUC 0.54–1.00). The discrepancies between cohorts may be attributed to the nonuniform geographical distribution, which affects the microbial communities [49].

Compared with the concrete composition, metabolites of microbiota may be more functionally meaningful, playing non-negligible roles in host immunity when absorbed into the blood system. Short-chain fatty acids (SCFAs), one type of microbiota metabolites in the gut, are known for their function in T cell homeostasis [51]. An investigation including 52 patients with solid tumors on nivolumab or pembrolizumab therapy demonstrated that Rs had higher levels of fecal and serum SCFAs pre-treatment compared with NRs (*P* < 0.05) [50]. Inversely, in another pooled dataset of patients with multiple myeloma treated with ipilimumab (*n* = 85), patients were classified into two subgroups based on median serum SCFA concentrations at baseline, and Kaplan–Meier analyses showed that lower levels of both butyrate and propionate had associations with longer PFS (*P* = 0.0015; *P* = 0.0029) [51].

These studies on the profiles of gut microbiota composition and metabolites in patients with cancer confirm their promising value in ICI efficacy prediction with a completely non-invasive approach. However, the effects of microbiome composition and SCFA-focused microbial metabolites on host immunity modulation are fairly intricate. Importantly, the microbial composition can be affected by geographic location [135], diets, intake of drugs (especially antibiotics) [136, 137], and lifestyle. Moreover, different cancer types, treatment regimens (single or combined therapy) [138], clinical response annotations, and bioinformatics methods all may contribute to significant inter-cohort heterogeneity and inconsistent results obtained at different institutions. Hence, a more comprehensive evaluation of microbial function and interactions with the host during ICI treatment is required. Furthermore, machine learning algorithms should be efficiently utilized for in-depth studies in larger cohorts to identify microbial biomarkers with excellent predictive performance.

### eNose-related biomarkers

As an emerging and completely non-invasive method for medical testing, with artificial intelligence, the electronic nose (eNose) can capture and classify the volatile organic compound in exhaled breath. The detection of epidermal growth factor receptor mutation with high accuracy in lung cancer provides compelling evidence for its efficacy [139]. Thus, some investigators speculated that molecular profiles of exhaled breath could reflect the inflammatory environment linked with response to anti-PD-1 therapy for patients within NSCLC [52]. They found that baseline data from eNose significantly differentiated responses at 3 months after treatment (training, *n* = 92, AUC 0.89, CI 0.82–0.96; validation, *n* = 51, AUC 0.85, CI 0.75–0.96). In addition, Buma et al. [53] confirmed the precise performance of eNose in distinguishing objective Rs at the early stage in NSCLC (*n* = 62 and 32, AUC 0.95 and 0.97, for training and validation sets respectively). Overall, the eNose is simple and easy to implement, after robust validation of accuracy in larger cohorts, this technology is promising to be used in clinical practice.

### Other biomarkers related to gender and body composition

At present, studies on biomarkers for ICI efficacy mostly focus on tumor mutation, antigen burden, and TME; however, many other factors related to clinical characteristics of patients such as gender and body mass index (BMI) may also have profound effects on the immune response. Differences in immune function between men and women exist due to genetics, hormones, and other factors [140]. A meta-analysis of 11,351 patients with advanced or metastatic tumors on ICI therapy [54] showed in comparison with the control group, the OS HR was 0.72 for men and 0.86 for women (95% CI 0.65–0.79; 0.79–0.93). The different ICI outcomes in men and women (*P* = 0.0019) indicated that in the era of precision medicine, more attention demands to be paid to gender heterogeneity and the promotion of the effectiveness of immunotherapy in women; ultimately, optimal and personalized therapeutic regimens for men and women will be explored.

Obesity has been reported to shape the metabolism in the TME, impair T cell infiltration and function, and lead to immune senescence and dysfunction by adipocyte-derived molecules (e.g., adipokines, hormones, cytokines), which can be reversed by anti-PD-L1 [141–143]. Studies have shown that obesity correlates with improved efficacy of PD-L1 blockade in both tumor-bearing mice and patients with malignancies [141]. A study explored the relationship between BMI and the efficacy of different therapies in metastatic melanoma [55]. They observed that, in the immunotherapy cohort, obese patients had more favorable PFS and OS in contrast to those with normal BMI (*n* = 207 and *n* = 331; HR 0.75, 95% CI 0.56–1.00 for PFS; HR 0.64, 95% CI 0.47–0.86 for OS). Interestingly, when further grouped by gender, this relationship was observed in men but not women. However, some other studies on ICI therapy in patients with melanoma did not demonstrate notable associations between BMI and superior survival outcomes [56]. Given that studies linking obesity to immunotherapy efficacy lack consistency and reproducibility, Young et al. focused on specific body components (muscle, fat, etc.) in patients with metastatic melanoma undergoing ICI therapy (*n* = 287) [57]. They found that patients with sarcopenic obesity showed shorter PFS (HR 1.4, *P* = 0.04) in univariable analyses. In multivariable analyses, those with a high total adipose tissue index had shorter PFS (HR 1.7, *P* = 0.04), which was especially evident in women (HR 2.1, *P* = 0.03). Patients achieving the best outcomes were characterized by high skeletal muscle gauge and intermediate total adipose tissue index (PFS and OS, *P* = 0.02). Most recently, researchers proposed that visceral adiposity and systemic inflammation are crucial prognostic indicators of ICI therapy in melanoma [144]. Taken together, these results highlight the role of body composition (including obesity, BMI, and more specifically, sarcopenic obesity or visceral adiposity) in tumor development and ICI treatment efficacy prediction.

## Conclusions

In summary, ICIs have shown amazing efficacy in cancer treatment; however, few patients achieve durable clinical remissions [145]. Currently, biomarkers approved for clinical decision-making of immunotherapy are mainly based on invasive surgery or tissue biopsy. They are not able to overcome the temporal and spatial heterogeneity and potentially bring a series of complications due to the operation. In this article, we provide a relatively comprehensive discussion of non-invasive predictive biomarkers for ICI efficacy from the perspective of recent advances in diverse fields. Specifically, markers developed by the combination of AI and radiomics may not only be regarded as alternatives to PD-L1 [22] but also outperform the existing Response Evaluation Criteria in Solid Tumors (RECIST) criteria for identifying long-term beneficiaries [146]. Based on PET/CT imaging of radiotracers, the systemic tumor and immune landscapes can be non-invasively visualized [147]. Surprisingly, LB can evaluate the interaction of tumor and host in cellular and molecular dimensions and may be an economical and easy-to-apply detection method. Research on predictive biomarkers related to gut microbiota and its metabolites for the efficacy of immunotherapy has been developing rapidly; nevertheless, heterogeneity across cohorts exists due to the influence of host diets, lifestyle, medication, and geographical distribution. Furthermore, by analyzing the composition of exhaled breath, the novel and non-invasive eNose technology may have a broader application beyond lung cancer [148]. Additionally, other clinical factors related to gender and body composition are readily available and should not be ignored in the era of precision medicine.

As described above, studies on ICI therapy have shown promising prospects for non-invasive biomarkers. Nevertheless, there are still challenges before they can be routinely implemented in medical practice and additional efforts could be made from the following aspects. To achieve widespread application, machine learning in larger datasets is required and more optimized algorithms should be developed. Importantly, multi-center randomized controlled trials with large cohorts are necessary for the identification and validation of biomarkers with robust and reliable predictive performance. Moreover, to achieve precise prediction, biomarkers applicable to various tumor types (specific subtypes or pan-cancers) should be identified. Lastly, the cost of detection should be minimized, enabling economical and extensive implementations of the non-invasive biomarkers in clinical practice.

Notably, owing to the performance limitation of a single biomarker, integrative models incorporating multiple biomarkers related to tumor-host interactions are required to predict ICI efficacy accurately and timely [149]. In the future, the identification of additional non-invasive and dynamically predictive biomarkers with high sensitivity and specificity is expected. Developed by different detection methods, these markers will be precisely implemented for application in populations with diverse disease states, helping identify those possible to derive the most benefit from ICI therapy and better guide treatment decisions for patients with tumors in clinical practice, which will ultimately contribute to the improvement of therapeutic efficacy in cancer and promotion of social and medical effectiveness.

## Data Availability

Not applicable.

## Abbreviations

ICI: Immune checkpoint inhibitor
PD-1: Programmed cell death-1
PD-L1: Programmed cell death-ligand 1
TME: Tumor microenvironment
TMB: Tumor mutation burden
LB: Liquid biopsy
AI: Artificial intelligence
FDG: Fluorodeoxyglucose
PET: Positron emission tomography
CT: Computed tomography
NSCLC: Non-small cell lung cancer
AUC: Area under the receiver operating characteristic curve
CI: Confidence interval
DLS: Deeply learned score
PFS: Progression-free survival
OS: Overall survival
DCB: Durable clinical benefit
GBM: Glioblastoma
rADC: Relative Apparent diffusion coefficient
HR: Hazard ratio
TILs: Tumor-infiltrating lymphocytes
^89^Zr: Zirconium-89
LAG: Lymphocyte-activation gene
^68^ Ga: Gallium-68
SUVmax: Maximum Standardized uptake values
CTCs: Circulating tumor cells
cfDNA: Cell-free DNA
ctDNA: Circulating tumor DNA
Rs: Responders
NRs: Non-responders
T_ex_ cells: Exhausted T cells
Tregs: Regulatory T cells
MDSCs: Myeloid-derived suppressor cells
TMR: Ratio of Tregs to Lox-1 + PMN-MDSCs
LIPS: Signature of the liquid immune profile
TCR: T cell receptor
tTMB: Tissue-based tumor mutation burden
bTMB: Blood-based tumor mutation burden
ORR: Objective response rate
GIN: Genomic instability number
eQTM: Expression Quantitative trait methylation
TCF7: Transcription factor 7
LIF: Leukemia inhibitory factor
HIC: Host immune classifier
HIC-H: HIC-Hot
HIC-C: HIC-Cold
AFP: Alpha-Fetoprotein
CRP: C-Reactive protein
CRAFITY: CRP and AFP in immunotherapy
HCC: Hepatocellular carcinoma
EV: Extracellular vesicle
SCFA: Short-chain fatty acids
eNose: Electronic Nose
BMI: Body mass index
